# Characterizing temporal and spatial recruitment of systemically administered RPE65-programmed bone marrow-derived cells to the retina in a mouse model of age-related macular degeneration

**DOI:** 10.1007/s00417-021-05358-y

**Published:** 2021-08-06

**Authors:** Carolina Francelin, Juliana Godoy, Xiaoping Qi, Juliete A. F. Silva, Maria B. Grant, Michael E. Boulton

**Affiliations:** 1 Department of Ophthalmology and Visual Sciences, University of Alabama At Birmingham, 1670 University Boulevard, Birmingham, AL 35233 USA; 2 Department of Hemotherapy and Cellular Therapy, Hospital Israelita Albert Einstein, São Paulo, SP Brazil

**Keywords:** Bone marrow-derived cells, Age-related macular degeneration, Retinal pigment epithelium, RPE65, Cell recruitment

## Abstract

**Purpose:**

Previously, we reported that the intravenous injection of bone marrow-derived cells (BMDC) infected with lentivirus expressing the human *RPE65* gene resulted in the programming of BMDC to promote visual recovery in a mouse model of age-related macular degeneration (AMD). The aim of this study was to characterize the spatial and temporal recruitment of these programmed BMDC to the retinal pigment epithelial (RPE) layer.

**Methods:**

C57BL/6J female mice received a subretinal injection of AAV1-SOD2 ribozyme to knock down (KD) superoxide dismutase 2 (SOD2) and induce AMD-like pathology. BMDC were isolated from GFP^+^ mice and infected with a lentivirus expressing *RPE65*. One month after SOD2 KD, fifty thousand GFP^+^ *RPE65*-BMDC were injected in the mouse tail vein. Animals were terminated at different time points up to 60 min following cell administration, and localization of GFP^+^ cells was determined by fluorescence microscopy of neural retina and RPE flat mounts and tissue sections.

**Results:**

GFP^+^ *RPE65*- BMDC were observed in SOD2 KD neural retina and RPE as early as 1 min following administration. With increasing time, the number of cells in the neural retina decreased, while those in the RPE increased. While the number of cells in peripheral and central retina remained similar at each time point, the number of BMDC recruited to the central RPE increased in a time-dependent manner up to a maximum by 60 min post administration. Immunohistochemistry of cross-sections of the RPE layer confirmed the incorporation of donor GFP^+^ BMDC into the RPE layer and that these GFP^+^ human *RPE65* expressing cells co-localized with murine RPE65. No GFP^+^ cells were observed in the neural retina or RPE layer of normal uninjured control eyes.

**Conclusions:**

Our study shows that systemically administered GFP^+^ *RPE65*-BMDC can reach the retina within minutes and that the majority of these BMDC are recruited to the injured RPE layer by 60 min post injection.

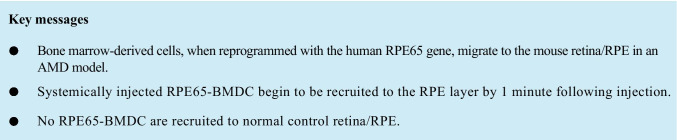

## Introduction

There is currently no reliable therapy for dry age-related macular degeneration (AMD). Two broad approaches, antioxidants and cellular therapy, have been utilized to address this major clinical problem affecting over 1.7 million in the USA [1]. One established cell therapy involves retinal pigmented epithelial (RPE) cell transplantation or replacement and represents a realistic strategy in the treatment of retinal degeneration [2, 3] as it involves one cell type and does not require the re-establishment of neural networks. Fresh and cultured RPE cells [2–4] as well as RPE cells derived from human embryonic stem cells or induced pluripotent stem cells have been utilized for subretinal transplantation [4–6], all with some success. While extremely promising, this approach does not overcome the necessity to invade the subretinal space, nor to disseminate cells across the fundus. An alternative approach to treating dry AMD is to use systemic delivery of an adult cell population that has the ability to home to dysfunctional RPE and to differentiate into the correct cell type. Bone marrow-derived cells (BMDC) offer just such an approach because they are an endogenous source of stem/progenitor cells, freely circulating throughout the body, and are easily removed and re-administered [7].

Previously, we reported that the intravenous injection of a subpopulation of BMDC infected with lentivirus expressing human *RPE65* were recruited to the neural retina and retinal pigment epithelial layer and promoted visual recovery in mouse models of AMD [8–10]. However, the spatial and temporal recruitment of these programmed cells to the RPE layer was not determined, and the earliest analyses of the donor cells were performed at 1 month following injection. Host bone marrow-derived cells, such as immune cells including BMDC progenitors, circulate throughout the mouse body and, if not recruited to injured tissues, will return to the bone marrow within minutes [11]. Thus, we proposed that RPE65-programmed BMDC will be rapidly recruited to the retina and begin incorporating into the RPE layer. Host BMDC such as monocytes can reach the retina and RPE via the retinal and ciliary body circulations [12–14]. Furthermore, experimental studies have demonstrated that a variety of cell types injected into the vitreous can successfully traverse the retina [15, 16]. In this study, we investigated the time course and spatial recruitment of systemically administered RPE65-programmed BMDC to the retina in a mouse model of age-related macular degeneration (AMD). BMDC were observed in the injured retina/RPE layer within minutes and appeared to be recruited via both the retinal and ciliary blood vessels.

## Methods

### Animals

All mouse experiments were conducted under protocols approved by the Institutional Animal Care and Use Committees at Indiana University (IACUC 11155) and University of Alabama at Birmingham (IACUC 20912) and in accordance with guidelines set forth by National Institutes of Health and the Statement for the Use of Animals in Ophthalmic and Visual Research of the Association for Research in Vision and Ophthalmology. Adult (8-week-old) female C57BL/6J mice and homozygous GFP transgenic (C57BL/6-Tg [UBC-GFP]) were purchased from Jackson Laboratories. At the end of the experiments, mice were euthanized by isoflurane in a desiccation chamber followed by cervical dislocation.

### Isolation and programming of BMDC

BMDC were isolated aseptically from tibiae and femurs of GFP transgenic mice as previously described [10]. Briefly, the bones were flushed using DMEM (high glucose) medium plus 10% fetal bovine serum (FBS) and 1% penicillin/streptomycin; red blood cells were lysed using ammonium-chloride-potassium (ACS) lysis buffer for 10 min. Cells were washed and using EasySep Mouse Hematopoietic Progenitor Cell Enrichment kit, the lineage negative (Lin^−^) cells were isolated. Lin^−^ cells were stained with Sca1-conjugated with PE for 40 min; Sca1^+^ cells were isolated by sorting using BD FACSAria. Lin^−^Sca1^+^ cells were resuspended in DMEM plus FBS and polybrene (4 μg/ml) to a final concentration of 5 × 10^4^ cells/ml. The cells were programmed ex vivo by inserting a stable human *RPE65* transgene using a lentiviral vector; 1 × 10^5^ cells were infected with 2 μL of lentivirus expressing TYF-RPE65 at a multiplicity of infection of ~ 50. Cells were centrifuged at 150 g for 2 h at 21 °C followed by washing and resuspension in sterile PBS for injection. The cell viability following spinoculation was 96.36% for control BMDC and 97.27% for RPE65-BMDC as accessed by Trypan blue staining and counting with a hemocytometer.

### Generation of SOD2-KD mouse model

SOD2 KD in C57BL/6 J mice was achieved by a subretinal injection of 1 µL of 2.5 × 10^12^ particles/ml of a recombinant AAV1 construct consisting of a pTR-UF2 vector expressing SOD2-specific hammerhead ribozyme, Rz432, driven by RPE-specific VMD2 promoter (AAV1-Rz-SOD2) as previously described [9, 17]. The contralateral uninjured eyes were used as a negative control.

### Intravenous injection of programmed BMDC

SOD2 KD mice received an intravenous injection of 5 × 10^4^ GFP^+^ *RPE65*-programmed BMDC in 100 μL of sterile saline into the tail vein (*n* = 8–10 per group).

### Preparation of retina/RPE flat mounts

At the appropriate time after injection of cells, the eye was enucleated and immediately placed in 4% paraformaldehyde for 24 h at 4 °C. No more than 10 s elapsed between enucleation and placing the eye in fixative. Following fixation, the lens, vitreous, and cornea were carefully removed, and 4 incisions were made to facilitate flattening of the posterior segment. The neural retina was separated from the RPE/choroid/sclera, and both were flattened onto glass slides with ganglion cell layer and RPE facing upward respectively. Mounting medium (ProLong Diamond Antifade Mountant, Invitrogen P36961) was applied followed by a glass coverslip.

### Imaging and analysis of GFP^+^ BMDC in retina/RPE flat mounts

**Fig. 1:**
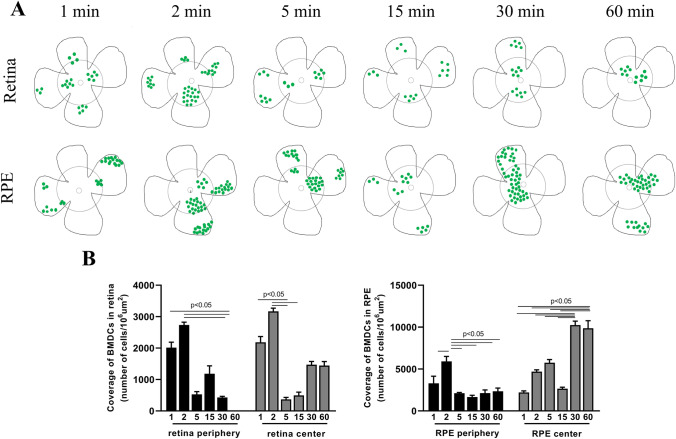
Spatial and temporal recruitment of GFP^+^ human *RPE65*-programmed BMDC to the neural retina and RPE. One month after AAV-SOD2 KD, mice received 5 × 10^4^ GFP^+^ RPE65-programmed BMDC via tail vein injection, and recruitment of these cells to the retina was determined over 60 min. (**A)** Diagrammatic representation of the appearance of donor BMDC in the peripheral and central (delineated by dotted line) neural retina and RPE layer at different time points. (**B)** Quantification of donor BMDC in central and peripheral RPE and neural retina at different time points. Values are expressed as the mean ± SEM and *p* < 0.05 is considered significant

Confocal imaging for endogenous GFP in GFP^+^ donor cells was performed using a Nikon A1R-HD25 Ti2 eclipse inverted confocal microscope in the UAB High Resolution Imaging Facility. All cells positive at 509 nm were considered to be GFP + donor BMDC. In some instances, flat mounts were also incubated overnight with anti-GFP (Abcam #ab13970) at 4 °C followed by Alexa-Fluor 488 (Invitrogen #A32723). Quantification of GFP^+^ BMDC in the retina and choroid/RPE flat mounts was performed in the central and peripheral fundus. Central was defined as 0.8 mm from the optic nerve head and the peripheral extended from this to the ora serrata (depicted as dotted line in Fig. 1). Fifteen images were taken from the peripheral and central areas under a 20 × objective from the total flat mounted retina in a masked fashion. Fifty GFP^+^ cells were counted on an average per retina using the cell counter tool from Image J software. Both eyes were analyzed, but the counting was performed only on the right eyes (SOD2 KD) as no GFP^+^ cells were observed in the contralateral uninjured eyes (control).

### Determination of GFP^+^ BMDC in retina/RPE sections

At the appropriate time after injection of cells, the eye was enucleated and immediately placed in 4% paraformaldehyde. No more than 10 s elapsed between enucleation and placing the eye in fixative. Following fixation, eyes were embedded in paraffin and 10 µm sections cut.

### Imaging and analysis of GFP^+^ BMDC in retina/RPE sections

Sections were deparaffinized and processed for antigen-epitope retrieval in a steamer at 120 °C for 45 min in antigen retrieval solution and then allowed to cool. Sections were then incubated with 5% normal goat serum (blocking solution) for 1 h. For double staining, the anti-RPE65 (Abcam # ab231782) and anti-GFP (Abcam #ab13970) were added to the slides and incubated overnight at 4 °C. After washing, slides were incubated with secondary antibodies Alexa Fluor 594 (Invitrogen #A21207) and Alexa-Fluor 488 (Invitrogen #A32723), respectively, for 1 h at room temperature. Slides were washed, and anti-fade reagent with DAPI (Molecular Probes by Life Technologies) was added. Confocal imaging was performed using a Nikon TE2000 inverted microscope (Nikon) at the UAB High Resolution Imaging Facility.

### Statistical analysis

For analyses, the investigator was blinded to the treatment groups. The cell counts are presented as mean ± SEM. Data were analyzed using GraphPad Prism Software. Statistical differences between groups were assessed by one-way ANOVA and the Kruskal–Wallis post hoc test. *P* values < 0.05 were considered significant.

## Results

**Fig. 2:**
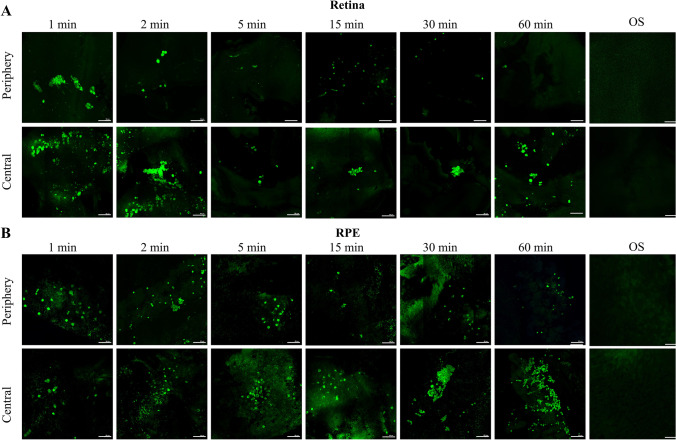
Time-dependent localization of GFP^+^ *RPE65*-programmed BMDC to the neural retina and RPE layer. One month after subretinal injection of AAV-SOD2 in mice to create an AMD-like phenotype, mice received 5 × 10^4^ GFP^+^ *RPE65*-programmed BMDC via tail vein injection. Recruitment of these cells to the retina was determined over 60 min. Representative fluorescence micrographs of central and peripheral retinal (**A**) and RPE/choroid (**B**) flat mounts at different time points show the localization of GFP^+^ cells. OS: contralateral uninjured eyes showing no GFP^+^ cells. Bar markers are 100 µm

RPE65-programmed GFP^+^ BMDC were observed in SOD2 KD retinas as early as 1 min post systemic injection (Fig. 1). At this time point, the GFP^+^ cells were observed throughout both the central and peripheral neural retina (4.2 × 10^3^ ± 7 × 10^2^ GFP^+^ cells/10^6^ um^2^). A similar number of cells were observed in the peripheral RPE layer (3.9 × 10^3^ ± 1.4 × 10^3^ GFP^+^ cells/10^6^ um^2^), but a significantly lower number were observed in the central RPE layer (2.2 × 10^3^ ± 3.7 × 10^2^ GFP^+^ cells/10^6^ um^2^). GFP^+^ cells were distributed randomly in the neural retina and central RPE, while in the peripheral RPE, GFP^+^ cells were localized predominantly as small groups of cells (Fig. 2A  and B). The highest number of GFP^+^ cells in the neural retina was observed at 2 min following injection (5.9 × 10^3^ ± 3.8 × 10^2^ GFP^+^ cells/10^6^ um^2^) with similar numbers of cells observed in the peripheral (6 × 10^3^ ± 1.5 × 10^3^ GFP^+^ cells/10^6^ um^2^) and central (5.7 × 10^3^ ± 7.4 × 10^2^ GFP^+^ cells/10^6^ um^2^) RPE (Figs. 1 & 2). At neither time points were GFP^+^ BMDC observed in the contralateral uninjured control eye.

Five minutes post injection, the number of cells declined by 2.8 and 6.5 times in the neural retina and peripheral RPE layer, respectively, and numbers remained constant thereafter out to termination of the experiment at 60 min with approximately 1.3 × 10^3^ ± 1 × 10^2^ GFP^+^ cells/10^6^ um^2^ in the neural retina and 2.3 × 10^3^ ± 7.8 × 10^2^ GFP^+^ cells/10^6^ um^2^ in the peripheral aspect of RPE (Figs. 1 & 2). By contrast, numbers of donor BMDC were highest in the central RPE at 30 and 60 min post injection reaching 1.0 × 10^4^ ± 9.5 × 10^2^ GFP^+^ cells/10^6^ um^2^ (Fig. 1 & 2). By 60 min, approximately 30% of the donor BMDC had been recruited to the retina/RPE. No GFP^+^ BMDC cells were observed to have been recruited to the contralateral uninjured control eye at any of the time points (Fig. 2).

**Fig. 3:**
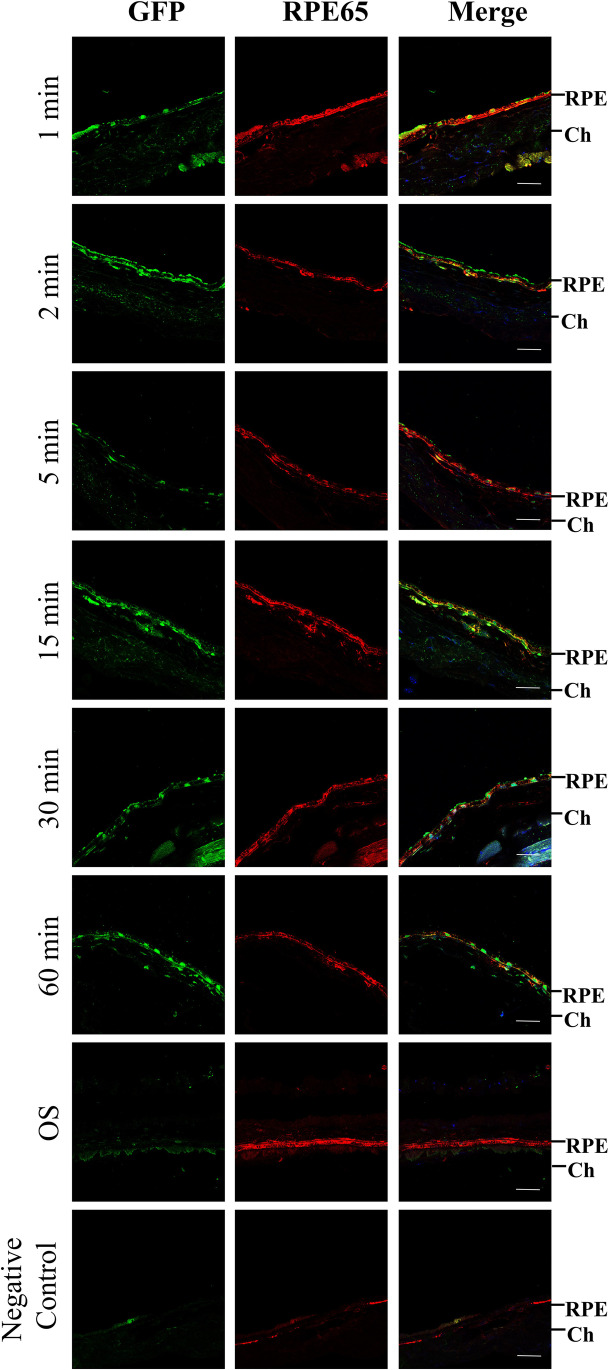
Dual immunostaining for GFP and human *RPE65* in cross-sections of the RPE layer. One month after mice received AAV- SOD2 via subretinal injection, mice received 5 × 10^4^ GFP^+^ *RPE65*-programmed BMDC via tail vein injection, and recruitment of these cells to the retina was determined over 60 min. Representative fluorescence micrographs of cross-sections of the RPE at different time points show the presence of GFP (green) and RPE65 (red) positive cells Co-localization of GFP^+^ and RPE65 by the same cell appears as orange in the merged figures. The nuclear stain for DAPI is blue. OS is the contralateral control eye showing no GFP staining in the RPE layer but strong RPE65 staining. The negative control is omission of the primary antibody. RPE, retinal pigment epithelium; Ch, choroid. Bar marker is 50 µm

Immunohistochemistry of cross sections of the RPE layer confirmed the incorporation of donor GFP^+^ BMDC in the RPE layer and these GFP^+^ cells co-localized with murine RPE65 (Fig. 3). These dual labelled cells were present as a few individual cells in the existing RPE layer at 1, 2, and 5 min following administration but appeared as small groups at the longer time points. No GFP^+^ cells were observed to have been recruited to the contralateral uninjured control eye, but we did observe typical RPE65 staining across the RPE layer (Fig. 3).

## Discussion

While we have previously demonstrated that systemic administration of *RPE65*-programmed BMDC are recruited to the injured RPE layer and sustain visual function in acute and chronic mouse models of retinal degeneration [8–10], we have not determined the short-term spatial and temporal recruitment of these cells to the retina. In this study, we show that programmed BMDC injected into the mouse tail vein are observed in both the neural retina and RPE by 1 min following administration with the number of GFP^+^ cells in the RPE increasing over a 60-min experimental time period.

The rapid recruitment of BMDC to the retina is not surprising given that: (1) the “one pass” circulation of blood in the mouse is around 15 to 20 s [18, 19]; (2) circulating monocytes/neutrophils are recruited to damaged tissue within an hour [20–22]; (3) intravitreal injection of BMDC results in their rapid incorporation into the retina and their migration to the RPE layer [23]; and (4) circulating BMDC are rapidly recruited to the retina in a variety of conditions [24–27]. Interestingly, the recruitment of programmed BMDC was only observed in the SOD2 KD eye with an AMD phenotype, and no GFP^+^ cells were observed in the fellow normal eye. This would suggest that the SOD2 retinas release inflammatory factors such as the cytokines CCL2 and CXCL12, which are known to attract monocytes to areas of tissue damage [20, 22, 28].

Spatial recruitment of the BMDC was apparent in both the central and peripheral retina. In the early stages, highest numbers of GFP^+^ cells were observed in the peripheral neural retina and RPE. However, this rapidly changed with the number of peripheral cells decreasing and a dramatic increase in GFP^+^ BMDC in the central retina with the majority of cells localized to the RPE layer. The origin of these cells is unclear but is likely to be from both the ciliary and retinal vessels as is the case for monocytes recruited to the retina [13, 26, 29, 30]. These observations are in line with previous reports demonstrating the migration of BMDC to the retina occurs via central retinal vessels, which express specific chemokines and cell adhesion molecules [13, 29]. Previous work has reported that BMDC have a region-specific preference in the retina to the ora serrata region [30]. The choroidal vascular route is less likely as the BMDC will not be able to cross an intact Bruch’s membrane. Future studies using real-time imaging will be necessary to fully confirm the vascular route by which these RPE65 programmed BMDC enter the retina. In our previous long-term studies [8–10], the donor GFP^+^ BMDC remained integrated in the RPE up to 6 months following systemic injection. However, donor BMDC were not observed in the neural retina at 1 month and longer following injection of RPE-65-programmed BMDC in this model indicating that any remaining cells will have migrated from the neural retina to the RPE layer. It should be noted that the RPE65-programmed BMDC will locate to other tissues/organs such as the spleen, lung, and bone marrow but that this is transient with all cells eliminated from these tissues by 1 month following administration [9].

## Conclusion

Taken together, our data show that the RPE65-programmed BMDC are rapidly recruited to the retina via the ciliary/inner retinal vascular and reach the RPE layer within minutes following systemic administration further supporting the feasibility of this approach for treatment of RPE pathologies such as AMD.

## Data Availability

All data has been included in the manuscript.
